# Nasopharyngeal Type-I Interferon for Immediately Available Prophylaxis Against Emerging Respiratory Viral Infections

**DOI:** 10.3389/fimmu.2021.660298

**Published:** 2021-05-19

**Authors:** Amos C. Lee, Yunjin Jeong, Sumin Lee, Haewook Jang, Allen Zheng, Sunghoon Kwon, John E. Repine

**Affiliations:** ^1^ Bio-MAX Institute, Seoul National University, Seoul, South Korea; ^2^ Department of Electrical and Computer Engineering, Seoul National University, Seoul, South Korea; ^3^ Interdisciplinary Program in Bioengineering, Seoul National University, Seoul, South Korea; ^4^ Department of Microbiology, Icahn School of Medicine at Mount Sinai, New York, NY, United States; ^5^ Institute of Entrepreneurial Bio Convergence, Seoul National University, Seoul, South Korea; ^6^ Seoul National University Hospital Biomedical Research Institute, Seoul National University Hospital, Seoul, South Korea; ^7^ Center for Medical Institute, Seoul National University Hospital, Seoul, South Korea; ^8^ Webb-Waring Center, University of Colorado School of Medicine, Aurora, CO, United States

**Keywords:** prophylaxis, emerging respiratory viral infection, type-I interferon, nasopharyngeal, post-pandemic, coronavirus, COVID-19

## Abstract

In addition to SARS-CoV-2 and its variants, emerging viruses that cause respiratory viral infections will continue to arise. Increasing evidence suggests a delayed, possibly suppressed, type 1 interferon (IFN-I) response occurs early during COVID-19 and other viral respiratory infections such as SARS and MERS. These observations prompt considering IFN-β as a prophylactic or early intervention for respiratory viral infections. A rationale for developing and testing intranasal interferon beta (IFN-β) as an immediately available intervention for new respiratory viral infections that will arise unexpectedly in the future is presented and supported by basic and clinical trial observations. IFN-β prophylaxis could limit the spread and consequences of an emerging respiratory viral infection in at-risk individuals while specific vaccines are being developed.

## Introduction

COVID-19 is in the spotlight now but new viral respiratory infections will likely, and regrettably, cause morbidity and mortality globally in the future (1). The clinical presentation following COVID-19 and other respiratory viral infections varies widely; however, many patients become critically ill and die. They frequently develop the acute respiratory distress syndrome (ARDS) and multiple organ failure (2)—disorders that require, but are not counteracted well by, ventilator support, high oxygen concentrations, extracorporeal membrane oxygenation, pharmacologic treatment, and/or extraordinary supportive care in intensive care units. These findings mandate considering ways to prevent the severe or fatal consequences of serious viral respiratory infections.

**Figure 1:**
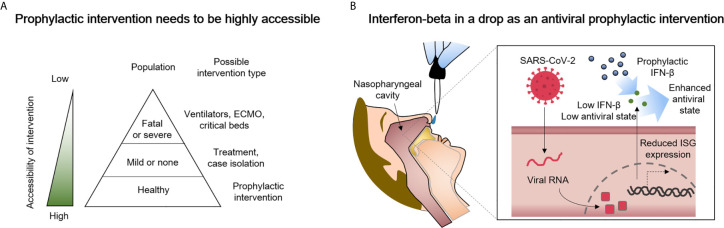
Delivery of type I interferon-beta (IFN-β) to the nasopharyngeal cavity is a candidate prophylactic and early intervention measure against COVID-19 that has high potential for success. **(A)** A greater accessibility of the intervention can prevent shortages of ventilators, extracorporeal membrane oxygenation (ECMO) machines, and/or critical care beds. **(B)** Suggested in this perspective is the highly accessible delivery of IFN-β to the nasopharyngeal cavity. The administered IFN-β can partially compensate for reduced interferon-stimulated gene (*ISG*) expression in SARS-CoV-2-infected cells as way to enhance antiviral immunity.

Despite laudable progress in treating respiratory viruses that was accelerated by efforts to combat COVID-19, when new viral pandemics emerge, shortages of beds, equipment, drugs, human resources, and specific vaccines will unfortunately again most likely contribute to poor patient outcomes. We anticipate a need for safe, prophylactic therapeutic strategies that can prevent or blunt the initial progression of future novel respiratory viral infections that will arise at times when existing improved therapies and vaccines may not provide complete, enduring, specific, and/or immediately accessible protection (**Figure 1A**). Virus induced impairment of innate immunity certainly facilitated the launch of the SARS-CoV-2 global pandemic that caused severe infection and death before proper therapies or specific vaccines were developed and tested. Regrettably, it could happen again with a new virus. In addition, a substantial number of individuals may choose to not become vaccinated and will remain susceptible. Accordingly, prophylaxis methods must be immediately available for the emerging respiratory viral infections, even though innate immunity can to some degree limit some viral diseases and delay severe infection or death before proper therapies or vaccine options are developed for the new diseases.

The possibility of administering IFN-I prophylactically to healthy individuals at higher risk of respiratory viral infections could have beneficial consequences. IFN-Is, which include IFN-α and IFN-β, are critical components of innate immunity and the initial cytokines produced by cells during viral infection. Because these interferons are produced during a viral infection, they undoubtedly play a major role in the initial innate antiviral immune response. As a frontline component of human immunity, IFN-1 is expressed by host cells in response to pathogens, induces expression of interferon-stimulated genes (ISGs), and initiates basic protective responses by immune cells. Since the first description of IFNs in 1957, IFN-1 has been suggested as a treatment for various viral and immune-related diseases, including multiple sclerosis (MS), viral hepatitis, and seasonal influenza.

The potential of IFN-1 as a treatment for viral respiratory infections is strongly suggested by the limited IFN-1 responses of SARS-CoV-2-infected patients (3, 4). The COVID-19 experience demonstrated that patients with SARS-CoV-2 are both (a) very susceptible to IFN-I and (b) uniquely adept at evading endogenous IFN-I by delaying or suppressing IFN-I expression (3–7). The ability of SARS-CoV-2 to inhibit endogenous IFN-I expression appears to make interferon pathway dysregulation critical to developing severe disease (5). Also, IFNs induce apoptosis of type II alveolar cells that are infected by viruses (6, 7). Moreover, when analyzing the transcriptomes, IFN-1 and IFN-3 levels are downregulated and innate antiviral responses are diminished in SARS-CoV-2 patients (8). Infection with SARS-CoV-2 produces a greater reduction in IFN-1 expression in host cells compared to the original severe acute respiratory syndrome coronavirus (SARS-CoV) (9). SARS-CoV-2 and SARS-CoV share over 90% amino acid identity but the proteins, which antagonize IFNs (nsp3, ORF3b, ORF6), have low sequence homology (10). ORF3b of SARS-CoV-2 has a premature stop codon that generates a truncated protein; ORF6b is missing two amino acids at the C-terminus and has decreased function. In addition, blood plasmacytoid dendritic cells from COVID-19 patients have impaired IFN-I generation (11). Collectively, SARS-CoV-2 can reduce IFN-I production by host cells and impair a protective early anti-viral response. Correcting these abnormalities could explain the enhanced susceptibility of SARS-CoV-2 to exogenous IFN-1 treatment. Not confining to SARS-CoV-2, the anti-interferon actions are observed in other respiratory infectious viral disease like Influenza A virus (12). Viral induced damage to the human respiratory, neurological, and other systems is often accompanied by IFN-I impairment (13).

We here describe a perspective on considering IFN-β as a potential prophylactic and early intervention measure against respiratory virus infection such as COVID-19 for healthy people (**Figure 1B**). The biology of IFN-β and pathology of SARS-CoV-2 are briefly described, several ongoing clinical trials are reviewed (**Table 1**), and the need for clinical trials based on the preventive use of IFN-β emphasized as they relate to future respiratory viral infections. Also, because the use of IFN-I may cause negative as well as positive effects, we describe previously reported adverse effects that the IFN-I may cause. IFN-I has long been used clinically, and we suspect that the nasopharyngeal delivery of IFN-I will have less adverse effects than the widely used subcutaneous doses.

**Table 1:** Clinical trials and results of IFN treatment.

Disease	IFN-type	Participants	Delivery method	Effect	Side effect	Reference
**COVID-19**	IFN-α	2944	Nasal drop	+	None	(14)
**COVID-19**	IFN-β-1b	127	Subcutaneous	+	None	(15)
**SARS**	IFN-α	190	Subcutaneous	–	None	(16)
**MERS**	IFN-α-2a	44	Subcutaneous pegylated IFN	+	None	(17)
**MERS**	IFN-α-2b	2	Subcutaneous pegylated IFN	+	None	(18)
**MERS**	IFN-α-2b	6	Subcutaneous	+	None	(19)
**MERS**	IFN-α-2b	5	Subcutaneous	–	Hard to define	(20)
**MERS**	IFN-α, β	51	Subcutaneous	+	Not reported	(21)
**MERS**	IFN-α-2a, β-1a	24	Subcutaneous	+	Not reported	(22)
**MERS**	IFN-α-2a, β-1a	11	Subcutaneous	+	Not reported	(23)
**MERS**	IFN-α-2b	2	Subcutaneous pegylated IFN	+	Not reported	(24)
**MERS**	IFN-α-2a	1	Subcutaneous	+	Not reported	(25)
**MERS**	IFN-α-2a	1	Subcutaneous	–	Not reported	(26)
**Multiple sclerosis**	IFN-β-1b	2220	Subcutaneous	+	Flu-like symptoms and abnormalities in liver function	(27)
**Multiple sclerosis**	IFN-β-1a	1106	Subcutaneous	+	Influenza-like illness, injection-site reactions, thyroid disorders, hepatic disorders	(28)
**Multiple sclerosis**	IFN-β-1a	383	Subcutaneous	+	Depression, influenza-like syndrome	(29)
**Multiple sclerosis**	IFN-β-1b	338	Subcutaneous	+	Not reported	(30)
**Multiple sclerosis**	IFN-β-1a, 1b	188	Subcutaneous	+	Not reported	(31)

## Intranasal IFN-β Administration May Be a Desirable Option for Initially Treating Emerging Respiratory Virus Infections

Intranasal administration of IFN-I is attractive since respiratory viruses infect humans through the nasopharynx which initially harbors high viral loads (32–34). Intranasal administration of IFN-α was reported to reduce seasonal influenza A virus morbidity in ferrets (35). Similarly, intranasal administration of IFN-I was shown to be effective in humans for prophylaxis against COVID-19. When recombinant human IFN-α was given as intranasal drops 4 times/day for 28 days to 2,944 (2,415 in the low-risk group, 529 in the high-risk group) healthy medical staff members, no symptomatic SARS-CoV-2 infection or adverse effects were reported in either group, while comparable hospitals had ~10% infection rate (14). Although not an official clinical trial, this observation offers promise that intranasal IFN-I prophylaxis could be effective when administered during the early stages of viral infection.

Because it is difficult to predict which antiviral will be best for prophylaxis of future respiratory viruses, extensive investigation of IFN-β, by which the exogenous administration seems to be more effective in inducing antiviral effect than IFN- α, is important to conduct now. IFN-β has been shown to induce stronger antiviral activity than IFN- α and is likely to be a more effective antiviral candidate than IFN-α for inhibiting coronaviruses (5, 22, 36–38). A recent clinical study of IFN-β as a prophylaxis against COVID-19 yielded meaningful results. On July 20, 2020, Synairgen plc (Southampton, United Kingdom) announced phase II results on 101 patients showing the odds of developing severe COVID-19 are reduced by 79% in patients receiving inhaled IFN-β treatment compared to placebo treatment. Moreover, COVID-19 patients receiving nebulized IFN-β are more than twice as likely to recover (39). Furthermore, rare genetic variants in TLR7 that impair *IFNB1* upregulation were identified in young men who developed severe COVID-19 (40). Despite the rising appreciation of the antagonism of IFN-I by SARS-CoV-2 (41), little investigation is being conducted to determine whether intranasal IFN-I can provide effective prophylaxis against SARS-CoV-2 and other respiratory viral infections. Ideally, these efforts could prospectively resolve manufacturing, pharmacokinetic, safety, efficacy, and other issues before a serious virus becomes widespread.

However, it is important that IFN-I should be used cautiously. For example, administration of IFN-I in high doses to patients may enhance cytokine storm and immunopathology. Especially, some of the side-effects previously reported in subcutaneously injected IFN-β include flu-like symptoms, depression, thyroid dysfunction, liver enzymes abnormalities, skin-site reactions, immediate post-injection reaction, lipoatrophy, hair loss, cardiotoxicity, and fatigue (42). However, these are reported in the clinical trials for treating MS and are injected subcutaneously. In clinical trials for treating viral infectious diseases like COVID-19, SARS, or MERS, little adverse effects have been reported (**Table 1**). Although more studies need to be conducted to why there are less adverse effects reported in viral infectious diseases, we suspect that the low levels of IFN-β in severe patients infected by coronaviruses have an effect (43). Therefore, because safety is the most important parameter for prophylaxis, we propose that IFN-β prophylaxis should be used by healthy people for preventing infections from emerging respiratory viruses to minimize the adverse effects of the IFN-β prophylaxis.

The fact that IFN-β appears to have little adverse effects for the respiratory infections in the literature (14–21, 23–31) (**Table 1**) is an important factor in selecting and preparing a candidate for emerging respiratory viral infectious diseases. Since IFN-β may provide its greatest impact as a preventive measure in at-risk individuals, the safety of IFN-β is important factor in selecting a candidate for treating emerging respiratory viral infectious diseases. Like in the COVID-19 pandemic, when a new respiratory viral infection emerges, options for emergency treatment are selected from the best available previously used drugs. IFN-β is not only already used for treating respiratory viral infections, but also currently being tested for use by pregnant women to treat COVID-19. IFN-β is assigned to a pregnancy category while most of the currently available treatment or vaccines are not (44). There were concerns in safety raised by the overexpression of the angiotensin-converting enzyme 2 (ACE2), the receptor in humans for virus entry for COVID-19. However, this concern was attenuated by reports that interferons induce a novel truncated ACE2 isoform, instead of the full-length receptor (45). Together, with the fact that IFN-β has long been proposed for immune-related diseases as well as the respiratory viral infectious diseases, IFN-β will also be a good temporary immediately available prophylactic counter measure for viral respiratory infections.

## IFN-β Needs to be Evaluated as an Intranasal Administered Prophylactic or Early Intervention Against Respiratory Viruses

Developing a formulation of IFN-β to counter the high viral loads when they are invading the intranasal mucosa is rationale (46). Intranasal delivery could be relatively more effective than systemic injections (47, 48). Lower IFN-β doses administered with a local or topical application can provide a relatively high drug concentration in a key location (49). In a recent clinical study by Synairgen, IFN-β treatment of chronic obstruction pulmonary disease patients aged 60 or higher showed very little adverse effects, substantiating that using IFN-β in high dosages might provide a safe prophylactic solution for preventing a viral respiratory infection. Considering the high viral loads that manifest early in the nasopharyngeal cavity, rigorous investigation to determine the value of delivering IFN-β directly into the nose should be undertaken. Some examples of intranasal delivery include nasal drops, nasal sprays, and nasal creams. The biophysical stability and manufacturing cost of IFN-β must be overcome but overcoming these and other hurdles will be worthwhile (50). Subsequently, clinical trials of IFN-β as a prophylactic and early intervention for respiratory viruses should be performed to determine if this approach could counter the rapid spread and reduce the consequences of a highly infectious virus. In addition to social isolation and masking, treating individuals with IFN-β could alleviate their symptoms, reduce the likelihood of the progression to more severe disease and, most importantly, reduce transmission.

## IFN-β Treatment Responses May Differ According to Disease Severity and Host Immune Status

IFN-β might be therapeutically effective in patients with severe symptoms but the greatest value might derive from early treatment of relatively asymptomatic individuals. Brzoska and his associates suggested that IFN-β1a should be provided to patients with severe symptoms due to coronavirus infections (49). In August 2020, a clinical trial sponsored by the National Institute of Allergy and Infectious Diseases (NIAID) began testing a combination of remdesivir and IFN-β in patients with laboratory-confirmed COVID-19 with evidence of lung involvement and severe symptoms. However, IFN-β has pro-inflammatory effects and may be contraindicated for treating patients with severe COVID-19 since robust IFN-β responses may occur in patients with severe COVID-19 (3, 51–53). A hyperinflammatory signature, with strongly upregulated expression of TNFα- and IL-1-driven inflammatory responses and vigorous IFN-1 responses, occurs in patients with severe, but not mild, COVID-19, suggesting that IFN-1 might accelerate progression from mild to severe disease by stimulating inflammation (11, 52, 53). Consequently, for patients who are in the later stages of COVID-19 with severe symptoms and/or organ damage, IFN-β may not be preferable to other treatments. Patients with severe symptoms can also develop autoimmunity with IFN-α although this rarely occurs with IFN-β (54). IFN-I treatment delivered *via* nebulizers produced better results as an early rather than an late stage intervention (55). Comparing the clinical outcomes of IFN-β treated patients with mild and severe viral respiratory infection should preferentially be performed only after evaluating prophylactic or early IFN-β treatment.

## Conclusion

Mutants of SARS-CoV-2 have recently been recognized (56). In spite of the controversy regarding the efficacy against the SARS-CoV-2 variants of the newly created vaccines, the lessons learned from the COVID-19 pandemic underscore the need to be better prepared for emerging respiratory infectious viruses. Although intranasal prophylaxis of IFN-β has neither proven safety nor efficacy in healthy population, it may be a universal solution for emerging respiratory viral infections. Treating healthy people with IFN-β during the initial phases of a viral infection outbreak could enhance protection beyond that afforded by mucosal immunity, physical isolation, and masks alone. Employing multiple interventions to prevent viral transmission is being promoted by the Imperial College COVID-19 Response Team to forestall and limit the overwhelming demands and ramifications caused by a pandemic respiratory viral infection (DOI: https://doi.org/10.25561/77482). It is reasonable to forecast that new respiratory infectious diseases will arise in the future and, accordingly, developing antiviral prophylaxis strategies now would provide a more favorable, timely, and prudent course of action while new specific vaccines and therapies are being developed (49).

## Data Availability Statement

The original contributions presented in the study are included in the article/supplementary material. Further inquiries can be directed to the corresponding authors.

## Author Contributions

AL, YJ, SL, HJ, AZ, SK, and JR designed the concept and wrote the manuscript. All authors contributed to the article and approved the submitted version.

## Funding

This work was supported by the National Research Foundation of Korea (NRF) grant funded by the Korea government (MSIT) (2020M3H1A1073304, NRF-2020R1A3B3079653).

## Conflict of Interest

The authors declare that the research was conducted in the absence of any commercial or financial relationships that could be construed as a potential conflict of interest.
